# From Electronegativity towards Reactivity—Searching for a Measure of Atomic Reactivity

**DOI:** 10.3390/molecules26123680

**Published:** 2021-06-16

**Authors:** Sture Nordholm

**Affiliations:** Department of Chemistry and Molecular Biology, The University of Gothenburg, SE-412 96 Göteborg, Sweden; ksjn@chem.gu.se

**Keywords:** atomic reactivity, electronegativity, atomization energy, hydride, G4 quantum chemistry, ionization energy, von Neumann ergodicity, ground state degeneracy

## Abstract

Pauling introduced the concept of electronegativity of an atom which has played an important role in understanding the polarity and ionic character of bonds between atoms. We set out to define a related concept of atomic reactivity in such a way that it can be quantified and used to predict the stability of covalent bonds in molecules. Guided by the early definition of electronegativity by Mulliken in terms of first ionization energies and Pauling in terms of bond energies, we propose corresponding definitions of atomic reactivity. The main goal of clearly distinguishing the inert gas atoms as nonreactive is fulfilled by three different proposed measures of atomic reactivity. The measure likely to be found most useful is based on the bond energies in atomic hydrides, which are related to atomic reactivities by a geometric average. The origin of the atomic reactivity is found in the symmetry of the atomic environment and related conservation laws which are also the origin of the shell structure of atoms and the periodic table. The reactive atoms are characterized by degenerate or nearly degenerate (several states of the same or nearly the same energy) ground states, while the inert atoms have nondegenerate ground states and no near-degeneracies. We show how to extend the use of the Aufbau model of atomic structure to qualitatively describe atomic reactivity in terms of ground state degeneracy. The symmetry and related conservation laws of atomic electron structures produce a strain (energy increase) in the structure, which we estimate by use of the Thomas-Fermi form of DFT implemented approximately with and without the symmetry and conservation constraints. This simplified and approximate analysis indicates that the total strain energy of an atom correlates strongly with the corresponding atomic reactivity measures but antibonding mechanisms prevent full conversion of strain relaxation to bonding.

## 1. Introduction

Linus Pauling was, for most chemists of the past century, instrumental in establishing the link between quantum mechanics and chemical bonding [1]. Particularly by his development of the valence bond (VB) theory he showed how chemical bonding and molecule formation could be seen as a consequence of the ground state orbital structure of the atoms. This structure, in turn, could be understood by chemists by reference to the periodic table and the Aufbau picture of energy-ordered one-electron orbitals. The rigorous implementation of the VB theory turned out to be difficult, but the simple empirical version was found to be very successful and is still heavily relied upon in the understanding and teaching of molecule formation in chemistry. While the great progress of quantum chemistry in predicting formation and properties of molecules has mainly been based on the Hartree-Fock independent electron theory (for a review of methodology see e.g., [2,3]), the VB approach has seen a recent revival in interest [4,5]. In the connection to development of the VB theory, Pauling also proposed that atoms could be assigned a property of “electronegativity”, the power of an atom in a molecule to attract electrons to itself, which could be relied upon to explain the direction and magnitude of the transfer of electrons when two atoms of different electronegativity EN formed a polar covalent bond [6]. He introduced a measure of EN determined from the assumption that the bond energy E_b_(AB) of atoms A and B would exceed the average of the bond energies E_b_(AA) and E_b_(BB) by an amount proportional to the square of the difference in electronegativity ΔEN = EN(A)-EN(B). This idea was a few years later taken up by Mulliken who proposed a measure for the electronegativity EN(A) directly from single atom properties, i.e., as the average of the first ionization energy and the electron affinity (defined as the first ionization energy of the negative ion A^−^) of the atom A [7].

Pauling’s concept of atomic electronegativity has, despite some lack of rigor and uniqueness as will be clear below, been accepted and made part of main stream chemistry [8,9,10]. Electronegativity is tabulated and used to predict and understand the appearance and stability of various types of bonds between atoms but exactly how to best define, calculate and interpret these numbers is still being discussed. It is generally agreed that the original statement of Pauling associating EN with an atom’s “power to attract electrons” applies but beyond that opinions vary. Some (e.g., [11]) emphasize that EN is not a fundamental atomic property but a property of an atom in a molecule. The Pauling definition of EN in terms of bond energies is consistent with this view but the Mulliken definition of EN certainly makes it a single atom property. Several other definitions of widespread use, e.g., the one by Allred and Rochow [12] in terms of an effective atomic charge and a covalent atomic radius and one by Allen [13] in terms of an average ionization potential for the valence shell of the atom, similarly suggest that EN is a single atom property.

It is beyond the scope of this report to review all interesting views, definitions, developments and interpretations of electronegativity. For a sample of these we refer to the recent literature [14,15,16,17,18,19,20]. Particular interest is drawn to the suggestion by Sanderson [21] that electronegativity can be regarded as a chemical potential which is equalized to some degree in the formation of molecules. This idea is of special interest in density functional theory of electronic structure [14,22]. Another interesting direction of progress is the “experimental” form of quantum chemistry proposed by Rahm and Hoffman [19,20,23,24], which also invokes ionization energies and electronegativity as key properties. Suffice it to summarize that the discussion has been, and continues to be, highly constructive and interesting even if not unified nor conclusive. It is not unreasonable to suggest that the ability to correlate electronegativity with, or determine it from, many different atomic or molecular properties has added value to the concept.

With this discussion of electronegativity in mind we shall now try to start, or at least contribute to, a discussion of the related concept of “atomic reactivity” AR. It is not the same as electronegativity. This is most easily seen by considering the inert gas atoms He, Ne, Ar, Kr, Xe and Rn. While generally overlooked, or deliberately not considered, in connection with electronegativity they play a central role in the determination of reactivity and should have well determined atomic reactivities AR of either zero or a local minimum in the dependence on the atomic number Z. These atoms are typically omitted from tables of electronegativities, although in some cases, like the Mulliken scale, they could readily be computed and are found to yield maxima in the periodic variation. For the noninert atoms, high EN is correlated with high reactivity but this rule is reversed for the inert gas atoms. Thus, a first objective of our concept and measure of atomic reactivity is to integrate inert and noninert atoms in the same scale consistently such that inert atoms are clearly seen to be so by representing local minima in the reactivity as a function of atomic number AR(Z).

There are more fundamental reasons why atomic reactivity is needed as a quantifiable property of atoms. This relates directly to chemical bonding and not only through the attraction of electrons to the atom but more generally through a change in electronic structure in the direction of that of an inert gas atom. It could again be argued, as in the case of electronegativity and perhaps with more reason, that reactivity is not a property of a single atom but of an “atom in a molecule”. The immediate defense is that this argument did not stop electronegativity of individual atomic species from becoming a highly interesting and practically useful concept. Moreover, the numbers that are obtained by various procedures and found in the tables certainly do associate a numerical electronegativity with a single atom, if not uniquely.

Unease may still linger. Given that “reactivity” generally implies bond formation with other atoms, it would seem even more apt to say that atomic reactivity is a property of an atom in a molecule, not of a single atom. Can “reactivity” be associated with anything but bonding? We shall argue that it can. We shall proceed on the premise that atomic reactivity does exist as a property of a single atom. This reactivity is drawn upon in molecule formation but only to the degree that bonding mechanisms allow. Both atomic reactivity and bonding are expected to be subtle concepts. Their unravelling may require an extended research effort over time, particularly in view of the diversity of molecules. Nevertheless, it is common practice to refer to atoms as reactive or inert and we merely seek a simple numerical implementation of the existing idea that individual atoms can be assigned an inherent reactivity. We are also fully prepared for the likelihood that such a reactivity will be incompletely, and to a greatly variable degree, expressed in any given bond or molecule the atom finds itself in. We are hoping to find the source of reactivity in the properties of a single atom and expect a measure of this source to make itself interestingly known in stabilities of molecules formed by chemical bonding. Thus we proceed, despite the lack of definitive proof, on the basis that atomic reactivity is a property of a single atomic species drawn upon in the formation of molecules.

There is reason for optimism that such a measure of atomic reactivity can be found. There is evidence that reactivity can be associated with symmetries and conservation laws applicable to the single atoms which “strain” (increase energy) and destabilize the ground states. We have earlier found [25] that the Thomas-Fermi (TF) theory [26,27], the earliest form of density functional theory (DFT) known to completely fail to reproduce chemical bonding [28,29], does so precisely because its semiclassical form provides an “inert gas model” omitting these strains in the electronic structure of the single atom. Thus one can, in principle at least, imagine doing quantum calculations with and without strain due to symmetry and conservation laws, for each atom identifying the cost in electronic energy of going from the inert gas model to the real constrained structure of the atom. This energy cost would then be the source of reactivity of the atom. Unfortunately, TF-DFT suffers from a number of other approximations and the failure to account for reactivity can only approximately be eliminated (see [22] Chapter 6 and [30]), but as we shall see below, it is still possible to get an idea of what atomic strain energies a rigorous analysis of this type would associate with dynamical constraints acting on electrons in atoms [31,32,33].

The strains of the atomic ground state structures discussed are related to what in classical mechanics would be referred to as “nonergodic effects”, i.e., effects related to the inability of dynamics to take the trajectory uniformly over all states of the given initial energy [34,35]. In quantum mechanics, such effects are associated with degeneracy or near-degeneracy of the energy levels in the spectrum of energy eigenstates and eigenvalues of the system [36,37], i.e., the atom in our case. Thus we can see clear indications of atomic reactivity already in the Aufbau picture of atoms, which displays degeneracies and allows near-degeneracies to be predicted. We shall show that this type of degeneracy analysis clearly identifies the inert gas atoms, with nondegenerate ground states, as nonreactive while all other atoms have degenerate and/or near-degenerate ground states indicating that they are reactive.

An outline of our report below is as follows. The main goal of this work is practical and the main analysis is empirical and exploratory. We therefore initially return to the original idea of electronegativity of Pauling [6], and the slightly reinterpreted form of electronegativity by Mulliken [7], to see if we can now use them to define and determine a property of “atomic reactivity”, which is closely related to “electronegativity” but is not the same. After a century of analysis and discussion of covalent bonding, we try below to define and extract numerical values for an atomic property that, more directly than electronegativity, identifies the reason in the quantum mechanics of atoms for chemical bonding and the source of the stabilization energy that we see in molecules and solid matter. We examine three different such measures inspired by the original work of Pauling and Mulliken. First, we reconsider the Mulliken electronegativity and define an atomic reactivity in terms of ionization potentials. We then follow Pauling and try to extract atomic reactivity from molecular stabilities in two different measures. The last of them, based on hydride bond energies and geometric averaging, will be our currently recommended measure, but we recognize a non-uniqueness as in the case of electronegativity. We then show how such atomic reactivity can be understood as a quantum mechanical consequence of conservation laws acting on the motion of the electrons in an atom. This is illustrated by the use of the familiar Aufbau picture of the electronic structure of atoms. The reactivity is found to be related to degeneracy and near-degeneracy of the ground state, which can be approximately resolved by the Aufbau rules. By reference to earlier work [32,33], it is also shown that the total strain energy of an atom can be semi-quantitatively estimated by use of the Thomas-Fermi theory, which in its initial form lacks the effects of conservation laws but can be extended to do so. The total strain energies of the atoms are found to be very much larger than the stabilization energies achieved in chemical bonding. This is seen as evidence for the presence of strong antibonding interactions between nuclei and between nonbonding electrons.

## 2. Search for an Empirical Measure of Atomic Reactivity

**Starting point:** Electronegativity EN is often perceived as connected to reactivity of atoms. Pauling [6] first extracted EN from diatomic bond energies, which must be related to atomic reactivity AR. However, atomic reactivities cannot be fully described by electronegativities EN. Two basic reasons for this are as follows:

(i) There are two main bonding mechanisms, ionic and covalent bonding. The electronegativity can only be directly related to the ionic mechanism and at best indirectly, or not at all, to the covalent bonding mechanism, which is more important.
(ii) The inert gas atoms are either trivially of an undefined negativity EN (since all bond strengths used in the Pauling measure vanish) or are alternatively found to be highly electronegative (EN(Z) are at periodic maxima for inert gases in the Mulliken measure and some other measures).

Our general approach is to use the famous definitions of electronegativity by Pauling and Mulliken as starting points for new empirical measures of AR obtained by revision of these traditional measures of electronegativity. Having obtained and examined such empirical measures, we then discuss the underlying origin of this reactivity in the strains imposed on atoms by quantum mechanics in the presence of conservation laws imposed on the dynamics of electrons in atoms.

**Mulliken approach:** The atom is A with atomic number Z. We expect high first ionization energy I_1_(A) to be associated with low reactivity and high electron affinity E_a_(A), defined as I_1_(A^−^), to be associated with high reactivity. Thus, we might anticipate reactivity to be proportional to E_a_(Z) − I_1_(Z) but E_a_(Z) is too small compared to I_1_(Z) due to charge imbalance in the negative ion created by the addition of an extra electron. Such charge imbalance does not occur in a neutral molecule. Any local charge imbalance is compensated by “screening” in some form which, due to the long range of the Coulomb potential, can be quite effective even at a considerable distance on a molecular scale. It seems therefore more realistic to work with neutral atoms in our search for a Mulliken-like atomic reactivity. Thus, by comparison with Mulliken’s electronegativity, we propose that in the atomic reactivity AR_M_ the term I_1_ appears with a sign change and the term E_a_ with a neutralization of the negative ion. Our proposed change measure from electronegativity EN to atomic reactivity in the Mulliken analysis AR_M_ is then as shown in Equation (1) below:

EN(A(Z)) = ξ (I_1_(Z) + E_a_(Z)) → AR_M_(A(Z)) = ξ(I_1_(Z + 1) − I_1_(Z)) (1)

Note that this reactivity measure AR_M_, like the original Mulliken electronegativity, is a single atom property which is proposed to be the source of a multitude of expressions of atomic reactivity in molecule formation. The anion A^−^(Z) is isoelectronic with the atom A(Z + 1) from which we obtain a measure of the stability of the negative ion, after neutralization in a molecule, in the form of I_1_(Z + 1). The prefactor ξ will here, with the reactivity on an energy scale, be taken to be ½ but can also be an inverse energy to make the measure dimensionless, e.g., to fix the reactivity of fluorine to be 4 by defining it as follows:

ξ = 4/(I_1_(10) − I_1_(9)) (2)

The choice ξ = ½ will make our reactivity measure an energy comparable to the corresponding electronegativity of Mulliken, while the latter choice in Equation (2) will provide a relative atomic reactivity that we may distinguish as rAR_M_(A). Note that the assignment of a relative reactivity 4 to fluorine is in accordance with common practice for electronegativity.

**Pauling approach:** Reactivity is, even more than electronegativity, directly related to molecule formation and stability. Instead of focusing, as did Pauling, on the contribution related to the difference (in EN) between bonded atoms, we take the view that bond energies are, to a first approximation, proportional to the sum of the reactivities of the constituent atoms:

E_b_(AB) ≈ ξ (AR(A) + AR(B) (3)

By this ansatz we can extract a simpler and more general reactivity measure which is applicable to the covalent form of reactivity.

Note that this simplest definition of atomic reactivity, assuming linearity in relation to bond energy, focuses exclusively on “covalent reactivity”, since it does not account for the ionic bonding mechanism drawn upon in the Pauling definition of electronegativity. It is easily seen that if (3) held precisely (with a constant ξ) then the Pauling electronegativities (also employing a linear average) would vanish. Clearly, an atomic reactivity extracted from (3) would be a “covalent reactivity”. The reality is, of course, that bonding arises by a range of different mechanisms including the basic covalent and ionic types. Moreover, these mechanisms are the subject of limitations in the form of antibonding mechanisms which will act with a strength dependent on the detailed molecular structure. Thus, our atomic reactivity cannot be expected to precisely satisfy (3) with a constant ξ but this relation will still, in the simplest analysis, identify the atomic source of bond stabilities in molecules. While there is clearly an interest in developing more refined measures of atomic reactivity than implied by relation (3), i.e., which accounts for both ionic and covalent bonding and, perhaps, resolves the different subtypes of covalent bonding, we shall start here with exploring the simplest type of covalent reactivity. Even so, linearity, as in relation (3), is not the only possible simple starting point, as we shall illustrate below.

**Reactivity from hydride atomization:** Atomic reactivity is expressed in terms of a wide range of molecules and bond types appearing in them. Even after accepting Equation (3) as the defining relationship for a Pauling-like measure of reactivity AR_P_, the way to extract numerical values is far from settled. The simplest separation of bond types is in the categories of ionic or covalent bonds. While the electronegativity measure relates most clearly to ionic bonding, the covalent bonding is more important in chemistry and we seek, as noted above, a reactivity measure primarily for covalent bonding. The relation (3) is, in its linearity, particularly suited to the covalent bonding mechanism. It is correspondingly unsuitable for the ionic bonding mechanism. The ionic bonding mechanism is inherently a binary atomic property ARI(A,B) requiring a relation going beyond linearity to at least bilinear terms in atomic reactivity. We recognize that the relation (3) will bias our measure of atomic reactivity towards its expression in purely covalent bonds. We accept that bias for the moment and return to reconsider it below.

The key question is now how to extract a single atom property AR_P_(A) from the great variety of bond energies E_b_(AB). It may seem logical to let a single atom property be extracted from the bonding of the atom to itself in A_2_, thereby avoiding a nonunique choice among atoms B, but we shall not do so. Drawing upon homogeneous diatomic molecules in this way would involve a variation of bond type with atom A in A_2_ that would seem certain to strongly influence the atomic reactivity obtained. We choose instead to let the reactivity be determined by the simplest bond type, making it more straightforward to determine and easier to understand. Among the many types of covalent bonds (e.g., single, double and triple bonds, polar or nonpolar bonds and further delocalized, or resonance enhanced bonds) we suggest that the single bond is the simplest, involving just one pair of shared electrons. Even single bonds are subject to mechanistic variation. If we exclude dative single bonds the remaining variation is due to what might be called Pauli repulsion, i.e., interactions between the nonbonding “other electrons”. These effects can be quite large. They are, e.g., generally assumed to be responsible for the otherwise unexpectedly weak bond in F_2_. We expect the fluorine atom F to be found to be very reactive, so we realize that the complication of Pauli repulsion should be reduced as far as possible. This leads us to consider single bonds to hydrogen, i.e., as in HF rather than F_2_.

In the range of all bonds, we claim, those of an atom A to a hydrogen atom enjoy a unique status as the simplest. The bond A-H is a single bond and it involves adding or removing a single electron or sharing a single electron pair. There are no nonbonding or antibonding electrons contributed by the hydrogen atom, so the expression of atomic reactivity in terms of chemical bonding involves a minimum of the complicating interactions of such electrons. In fact, hydrogenation serves as a simple way of sealing off the reactivity of atoms or molecular fragments. It is indeed used as a way to allow fragments of larger molecules to be studied independently and more efficiently by quantum chemical methods very sensitive to the size of the molecule (as in the link atom scheme used in the QM/MM method [36]).

We choose, therefore, to extract atomic reactivities from atomic hydrides AH_n_ with n chosen so that it is as small as possible but such that either A^n+^ or A^n−^ (or both) is of an inert gas structure, i.e., of electron number 2, 10, 18. We shall consider the first 20 atoms with atomic number Z = 1, 2, …20. The selected hydrides are H_2_, LiH, BeH_2_, BH_3_, CH_4_, NH_3_, H_2_O, HF, NaH, MgH_2_, AlH_3_, SiH_4_, PH_3_, H_2_S, HCl, KH and CaH_2_ with He, Ne and Ar taken not to bind hydrogen. These seventeen hydrides contain only single bonds and they minimize the role of nonbonding electrons and of more complex bonding mechanisms. Ionic bonding may make a significant contribution to the stability of LiH, BeH_2_, NaH, MgH_2_, H_2_O, HF, NaH, MgH_2_, HCl and KH, but the covalent contribution is likely to dominate even in these molecules. One may think of hydrogenation as nature’s simplest way of healing the atomic strain by providing neutralized radical electrons in the form of hydrogen atoms. It provides, we suggest, the easiest way for us to extract atomic reactivity from the greater complexity of bonding in molecules.

**Definition:** A new measure of atomic reactivity in terms of energy is proposed such that the reactivity of hydrogen AR_P_(H) is half the bond energy of H_2_ and the reactivity of other atoms obtained from the atomization energy E_at_ of the inert gas like hydride AH_n_, as defined above, by the relation:

AR_P_(A) = E_at_(AH_n_) − nAR_P_(H) = E_at_(AH_n_) − (n/2)E_at_(H_2_) (4)

The definition is meant to apply primarily in the normal quantum chemical simplification of temperature 0 K and infinite atomic masses, i.e., in the Born-Oppenheimer model of molecules. The reactivity is then entirely electronic but it can be extended to include effects arising from the nuclear motion with some attention to details of vibrational, rotational and translational modes and energies. It could then also be applied for real molecules with atoms of finite masses at a standard temperature. The changes due to nuclear motion will generally be small and likely be insignificant or negligible in most, but not all, cases. The largest effect is likely to come from the vibrational zero point energy of H_2_ and the hydrides which, e.g., if accounted for in the reactivity of H, would reduce it by up to about 10%. Nevertheless, it is reasonable to recognize but leave aside these “nuclear quantum effects” and use the simpler “electronic atomic reactivity” unless the application is specifically addressing isomeric shifts or phenomena specifically related to the nuclear motions.

**Implementation and Results**

The measures constructed above, AR_M_(A) based on first ionization energies and AR_P_(A) based on hydride atomization energies, have been implemented for the first 20 atoms for exploratory purposes. The energy unit used is kJ/mol. The ionization energies are from a standard table [37]. The atomization energies of atomic hydrides are quantum chemical and obtained by the Gaussian-4 (G4) method [38], chosen to obtain accurate and comparable results over the large range of atoms. The atomization energies are expected to be obtained within ±3.5 kJ/mol, which will normally provide bond energies and atomic reactivities accurate to at least two significant figures. We recall that the “most stable” hydrides chosen are H_2_, HeH, LiH, BeH_2_, BH_3_, CH_4_, NH_3_, H_2_O, HF, NeH, NaH, MgH_2_, AlH_3_, SiH_4_, PH_3_, H_2_S, HCl, ArH, KH and CaH_2_. The three inert gas hydrides are taken to be unstable, since we do not account for any weak complexes that may form by physical rather than chemical mechanisms. All other hydrides are stable.

Although the measures in energy are informative in their own right, we are also interested in the relative reactivities of atoms. In order to facilitate such a comparison we define, as previewed for the Mulliken measure above, a dimensionless relative atomic reactivity such as:

rAR(A) = 4 × AR(A)/AR(F)(5)

**Figure 1:**
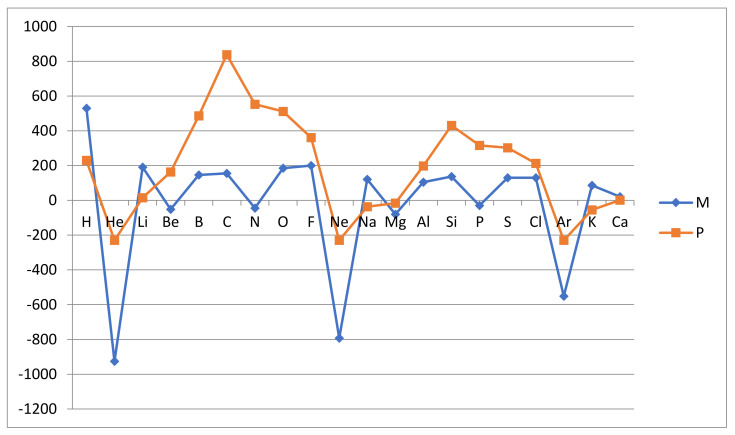
The atomic reactivities AR_M_(A) based on first ionization energies (diamonds) and AR_P_(A) based hydride atomization energies (squares) are shown in kJ/mol for 20 atoms from H to Ca.

**Figure 2:**
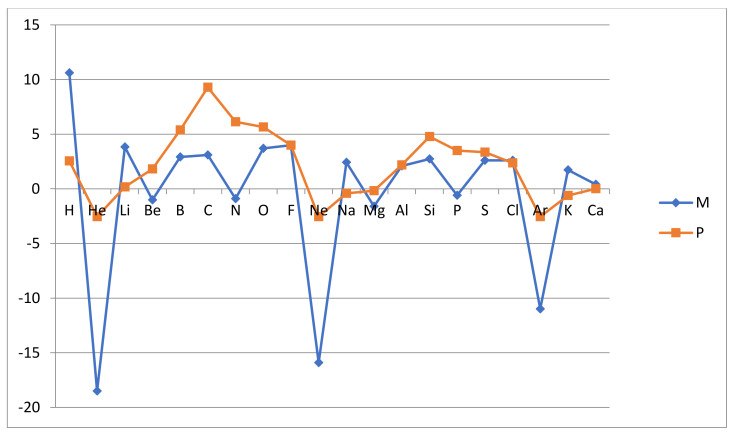
The relative atomic reactivities rAR_M_(A) and rAR_P_(A), normalized so that the value for fluorine is 4, are shown for atoms A from H to Ca.

Thus, the relative atomic reactivity (whether the Mulliken related rAR_M_ or the Pauling related rAR_P_) is normalized such that fluorine has the relative reactivity rAR(F) = 4 and the other atoms have corresponding relative reactivities anchored to the same scale. The results are collected in Table 1 below and are graphically illustrated in Figure 1 and Figure 2.

**Significant trends**: The results show most clearly that the inert gas atoms are nonreactive and in this sense quite distinct from the other atoms. This is dramatically evident in the AR_M_ measure, which is a forward derivative of the first ionization energy I_1_(Z) with respect to atomic number Z. The other (noninert) atoms are all reactive in the sense that they form stable hydrides and have, with one exception, at least one positive of the two reactivities AR_M_ and AR_P_. The exception is magnesium Mg, where both these reactivities are negative. There are some further noninert atoms which have one negative reactivity of the two, i.e., AR_M_(Be) = −51, AR_M_(N) = −45, AR_P_(Na) = −37, AR_M_(P) = −30 and AR_P_(K) = −55, all in kJ/mol. This may seem surprising, but we should note that for these measures our scale of atomic reactivity is not such that a negative number means nonreactive. Certainly, the most reactive atoms are expected to have positive AR_M_ and AR_P_ values but still may not, as is illustrated by nitrogen N. The reason for this will be revealed below when considering the different character of the two measures.

The two measures are in general agreement about the separation of the atoms into group I inert (He, Ne and Ar) and nonreactive and group NI noninert and reactive (H, Li, Be, B, C, N, O, F, Na, Mg, Al, Si, P, S, Cl, K and Ca). The ionization energy measure AR_M_ emphasizes the distinction more than the hydride stability measure AR_P_. The reason for this is that AR_M_ can describe how resistant the atom is to releasing or adding an electron, while AR_P_ simply records that no stable hydride forms and assigns a formal negative value to the reactivity. The inert gas atoms stand out as nonreactive in the Mulliken-like measure AR_M_ since in the defining relationship, eg. for helium

AR_M_(He) = ½ (I_1_(Li) − I_1_(He)) (6)

and corresponding expressions for the other inert gases, the first (Li) and second (He) ionization energies are at local periodic minimum and maximum, respectively.

Another significant deviation between the measures is seen around the shell midpoints where the L and M valence orbitals are half full. The measure AR_P_ shows a maximum at the period midpoint while AR_M_ is more flat in the period with a minor minimum at the midpoint. Thus, C and N are the most reactive L-shell atoms according to the hydride stability measure AR_P_, while according to the ionization energy measure AR_M_, N is very weakly reactive and C only moderately so. A similar pattern is seen for Si and P in the next row. This deviation between AR_M_ and AR_P_ is related to the fact that AR_M_ is a “one-electron measure”, while AR_P_ is a “one- up to four- electron measure”. In the Mulliken approach, we consider the energetics of removing or adding one electron, while in the Pauling approach we can account for the removal or addition of up to four electrons by the covalent bonding, to up to four hydrogen atoms. Thus, the ability to form multiple bonds is proportionally reflected in the AR_P_ measure, as defined in (3). This readily explains why C and N appear to be strongly reactive in the Pauling approach but only more mildly so in the Mulliken approach, which is focused on the strength of a single bond, thereby favoring F. We also note that both N with the Aufbau structure 1s^2^2s^2^2p^3^ and P with the structure 1s^2^2s^2^2p^3^3s^2^3p^3^ enjoy the particular stability of a half-filled np-subshell, which reduces the ionization energy estimate of reactivity AR_M_.

Finally, we also note that the variation of reactivity with the atomic number is largest in the first row of the L-shell atoms and similar but smaller in the second row of M-shell atoms. This is a well-known feature of chemical bonding, i.e., the weakening of the bonding mechanism as we move from the first to the second and higher periods, which is reproduced by our reactivity measures.

**Geometric average form of Pauling reactivity:** As is clear in the results above, the simplest linear relationship and corresponding algebraic average reactivity proposed in the Pauling approach above has an apparent disadvantage in that the inert gas atoms will be assigned formal negative reactivities, i.e., −230 kJ/mol, which is half the bond energy of H_2_ and is unrelated to the specific nature of the inert gas atom. This follows since in the linear proportionality (3) and the reaction of an inert gas atom with hydrogen, or other reactive atoms, the vanishing atomization energy means that positive reactivities of these atoms must be cancelled by negative reactivities of the inert gas atoms. Thus, the lack of reaction of an inert gas with a reactive atom influences the measured inert gas reactivity strongly, but assigns a reactivity determined only by the choice of the reference atom, i.e., hydrogen in our case.

We seek now a better measure, which would treat nonreactivity more realistically and hopefully thereby become less dependent on the choice of reference atom (H). One could eliminate the presence of negative reactivities AR_P_ by simply shifting the scale so that the following relationship applies:

AR_P_(A) = E_at_(AH_n_) − (n − 1)AR_P_(H) = E_at_(AH_n_) − ½(n − 1)E_at_(H_2_) (7)

Another alternative is to allow not only A-H bonding in the hydrides but also H-H. This would mean the inert gas hydride would be, e.g., HeH_2_, which would be interpreted as He loosely attached to H_2_. We would then arrive at vanishing reactivities for the inert gas atoms and the negative reactivities of Na, Mg and K would change to zero as well. The latter shift is worthy of note since it points out that for these three atoms, Na, Mg and K, the hydrides are unstable to formation of hydrogen gas and could be regarded as vehicles for hydrogen storage, which is a process of great technical interest [39].

We shall not pursue these more technical cures of the apparent flaw of the linear average approach to a Pauling-like atomic reactivity measure. They still leave unresolved the underlying problem that bond stability is bilinear (or possibly of higher complexity in larger molecules) in atomic reactivity. If one atom is completely nonreactive, it does not matter how reactive the other one is. The bond between them will still have zero strength. The simplest incorporation of this basic fact is to express the molecular stability as a sum of geometrically averaged atomic reactivities obtained from bond energies E_b_(AB). Thus, we define the new reactivities from the relation

E_at_(AH_n_) = n × E_av_(AH) = ξ × n × (AR_GP_(A) × AR_GP_(H))^½^ (8)

Here the average bond energy is obtained as

E_av_(AH) = E_at_(AH_n_)/n (9)

This geometric relation is inherently nonlinear in the expression of bond strength in terms of atomic reactivity. It is also a “single bond measure of atomic reactivity” and will therefore remove or reduce the influence of multiple bonding capacity on the atomic reactivity, which shifted reactivity AR_P_ to the centers of the periods (C and Si, respectively). The proposed modification based on bond energy as proportional to a geometric average of atomic reactivities, AR_GP_, will thereby become more compatible with the one-electron measure AR_M_(A). We will moreover find that all atomic reactivities AR_GP_(A) will be greater than zero, except for the inert gases for which they will be zero. Recall here that we do not account for weakly bound complexes that may form with inert gas atoms by attraction other than chemical bonding, e.g., dispersion and polarization.

The defining relation of our geometric Pauling (GP) type reactivity is:

AR_GP_(A) = [E_at_(AH_n_)]^2^/[2n^2^E_at_(H_2_)] (10)

Note that we have added the factor ξ = 2 in (8) in order to allow AR_GP_(H) to agree with AR_P_(H). We do the calculation in two steps:

E_av_(AH) = E_at_(AH_n_)/n, AR_GP_(A) = ½ × (E_av_(AH))^2^/E_b_(H_2_) (11)

The average bond energies are interesting in their own right. Finally, we obtain a relative reactivity with that of fluorine fixed at 4:

rAR_GP_(A) = 4 × AR_GP_(A)/AR_GP_(F) (12)

The results are shown in Table 2 below.

**Figure 3:**
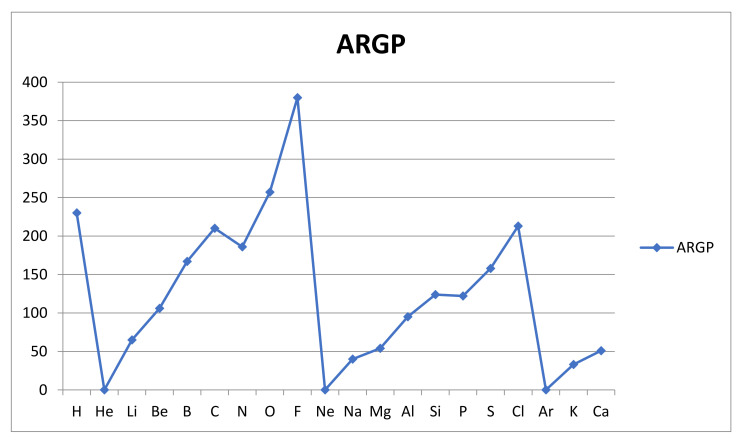
The atomic reactivity measure AR_GP_(A) in kJ/mol is shown for the atoms A from hydrogen to calcium.

The results for the reactivities AR_GP_(A) are shown graphically in Figure 3 below.

We note that the geometric average introduced into the bond energy-based measure AR_GP_ of atomic reactivity produces a very simple and plausible variation over the first 20 atoms, with its trends clearly recognizable from the variation in first ionization energy and electronegativity (the inert gas atoms omitted). The variation is clearly periodic, with minima at the inert gas atoms He, Ne and Ar followed by a steady rise, with an expected minor interruption at the half full valence np-subshells for nitrogen and phosphorous. The rise within a shell is clearly due to the rising charge of the nucleus, and the slight drop in reactivity as we go from the first to the second period is due to the increasingly peripheral nature of the valence shell, with the rising principal quantum number n, which is seen also in the first ionization energies.

**Figure 4:**
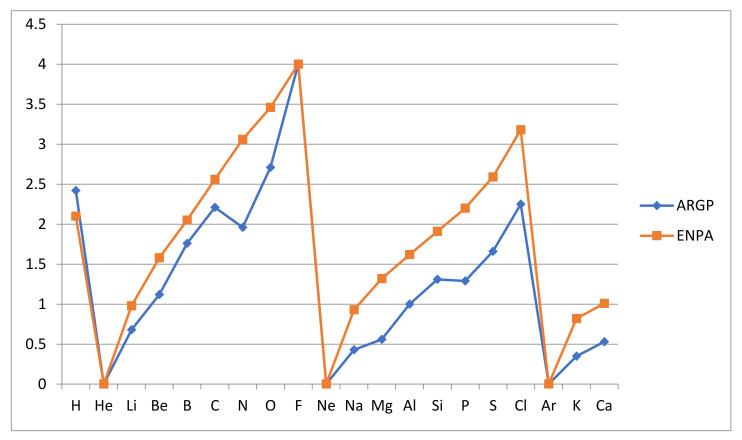
Comparison of relative reactivity rAR_GP_ (ARGP) and Pauling/Allred negativities (ENPA) for the atoms from hydrogen to calcium. Note that the electronegativities have been set to zero for He, Ne and Ar, while normally considered undefined for inert gas atoms.

Finally, it may be of interest to see the correlation between the relative atomic reactivities AR_GP_ and the corresponding electronegativities EN_PA_ as obtained by Pauling and refined by Allred [40]. They are compared in Figure 4 below. The apparent similarity is enhanced by taking the inert gas electronegativities to vanish. They are normally left undefined in the Pauling approach but appear as periodic maxima in the Mulliken approach to electronegativity. We can conclude that, as has been frequently assumed in practical use, the correlation between atomic reactivity and electronegativity is, if we avoid the issue with the inert gas atoms, very strong. This may surprise and call the need for the new concept atomic reactivity into question, but one need only imagine the comparison if the electronegativity values really obtained for the inert gases were inserted to realize that the opposite is closer to the truth. The reactivity measure enjoys a more secure, if so far less explored, foundation and graphical form. Still, the similarity of the trends within the periods is noteworthy.

Finally, before leaving this specific measure of atomic reactivity based on hydride atomization energies, we should consider whether there is any limitation waiting as we extend the measure beyond the first 20 atoms. No doubt there are cases where the choice of “most stable hydride”, which has been straightforward so far, will be less obvious. This might happen, e.g., due to underlying changes in structure relating to d-electrons. Relativistic corrections will become significant and affect electrons differently depending on the subshell *n, l* they are in. Nevertheless, there is no reason to expect the basic validity of the hydrogenation as a mechanism of relaxing strain in the electronic structure to falter. It is expected to remain reliable and useful, even for larger atoms of a more complex electronic structure.

## 3. The Quantum Mechanical Origin of Atomic Reactivity and Its Periodic Variation

We have above proposed three different empirical measures of atomic reactivity where the last one, based on single A-H bonds and geometric averaging, is likely to be particularly useful. Like Pauling, we have extracted this measure AR_GP_(A) from the contribution of this atom to the stability of molecules in which it participates. We now turn to the question of the fundamental origin in the electronic structure of single atoms of the reactivity as reflected in both ionization energies and molecular stabilities and the corresponding reactivity measures we have proposed.

The origin of the periodic variation in atomic reactivity seen in our results is clearly also the origin of the “shell structure” of the atoms, which is the basis of the periodic table. Every chemist knows that the Aufbau picture of electrons placed in shells (K, L, M … or *n* = 1, 2, 3 …), subshells (*n, l* with *l* = 0, … n − 1), orbitals (*n, l, m_l_* with *m_l_* = −l, …l) and spin orbitals (*n, l, m_l_, m_s_* with *m_s_* = ±½), which are energy ordered according to empirical “Aufbau rules”, explains and extends the periodic table, which in turn displays systematic trends for practically all atomic properties. These “periodic properties” most particularly and importantly include their reactivity. In fact, it could be fairly said that the periodic table has been a graphical organizer and predictor of atomic reactivity since its inception. Typically we have referred the question of an atom’s natural reactivity, and preference for different types of bonding in molecule or solid matter formation, to the periodic table. Thus, our effort to construct a measure of atomic reactivity is mainly in search of numbers of well understood meaning to gainfully extend the qualitative predictions obtainable from the periodic table.

After the introduction of quantum mechanics (QM) early last century, it has become clear that its laws contain within them the origin of these Aufbau rules and periodic variations of atomic properties seen in the periodic table. The shell structure of the atoms captured in the periodic table and Aufbau rules is known to be the result of the conservation laws applicable to the quantum dynamics, or equivalently a set of energy eigenstates, of the electrons in the spherically symmetric field of the atomic nucleus. Conservation of spin and orbital angular momentum, exactly for the total system of nucleus and electrons and approximately for each electron in the one-electron mean field (or SCF) model, results in a decomposition of the quantum energy eigenstates into degenerate (several states of identical energy) or near-degenerate clusters of states of small energy splittings. We argue below that the occupation of some, but not all, one-electron states of such clusters in the atomic ground state brings reactivity, while complete occupation, or none at all, is the hallmark of inert gas atoms without atomic reactivity.

It is a well-known fact of quantum mechanics that systems with widely separated energy states are comparatively resistant, while those with states of the same energy or small energy splittings are susceptible to coupling by external forces. This is seen, e.g., in the perturbation theory of external interactions [41], which requires special treatment (prediagonalization) of degenerate and near-degenerate states. The expansion coefficients showing the response of an energy state to the coupling in first order perturbation contains the energy splitting between coupled states in the denominator. The effect of the coupling is therefore boosted by multiplication with the inverse energy gap between coupled states. Conversely, the natural tendency of a system as it becomes well coupled (or inert) is to eliminate degeneracy and push energy levels apart (see reference [41]). Thus, independent atoms far apart, with filled and unfilled orbitals of the same or similar energy, are likely to be incompletely coupled in this sense and are therefore strongly susceptible to interaction as they approach and may then readily form molecular orbitals and stable molecules. The clustering of the highest occupied and lowest unoccupied atomic energy eigenstates is therefore a condition for reactivity, while well energy separated (or completely uncoupled) such states make the system inert. We shall look now at the causes and conditions for these cases to arise in atoms.

**Degeneracy and nonergodicity in electronic structure**: In order to expose the generality of the following analysis, we use the concept and terminology of “ergodicity”, which may be unfamiliar. We therefore introduce them first in classical Newtonian mechanics. For classical mechanics, the concept of ergodicity plays a key role in describing and understanding the qualitative character of the dynamics of a system. Very briefly, an ergodic system is, in classical dynamics, such that over time all states of the given energy of the trajectory (with all chosen starting points except a set of measure zero) will be visited (or approached) in proportion to its weight in the microcanonical ensemble at that energy [42]. Thus, the use of molecular dynamics to calculate microcanonical (fixed energy) averages of molecular systems is based on the assumption that the system is ergodic at the energy chosen. This assumption is referred to as “the ergodic hypothesis”. A nonergodic system, on the other hand, is “dynamically decomposable” such that the ensemble of all states of the given energy can be subdivided into parts between which dynamics cannot provide passage. While real systems of significant complexity are often implicitly assumed to be ergodic, simple model systems are often readily seen to be nonergodic. For example, all systems of only harmonic interactions are trivially seen to be nonergodic since they separate into independent normal modes of vibration and the energies of these modes are constants of the motion. In a simplistic language, one might say that an ergodic system is “fully coupled and connected”, while a nonergodic system is only “partially coupled and connected”. The possibility that an external interaction provides coupling and connection between previously disconnected parts is then the reason why a nonergodic system is likely to respond more strongly to external interaction and thereby be more “reactive” than an ergodic system.

Two questions now arise. Can this characterization in terms of ergodicity be generalized to quantum dynamics of electrons? If so, what role may it play in resolving atomic reactivity and its consequence in the form of bonding and molecule formation?

**The von Neumann form of quantum ergodicity—Relation to atomic reactivity:** von Neumann [43] suggested that in quantum dynamics the system was to be called “ergodic” if the spectrum of energy eigenstates was nondegenerate, i.e., to each energy eigenvalue one unique eigenstate would be attached. This would imply that nonergodicity would be reflected only in the degeneracy of the spectrum of energy eigenstates, i.e., nonergodicity at an energy E would mean that this energy corresponded to two or more eigenstates. Later [44], it was realized that quantum nonergodicity, or at least effects of a similar nature, could arise more generally, i.e., for nondegenerate energy eigenstates, when the system was dynamically decomposable, e.g., as in the case of separable motions of harmonic oscillators. Moreover, it was realized that nonergodicity could be seen in a “localization” of the energy eigenstates and in noisy gaps in energy between neighboring eigenstates in the complete spectrum and, in particular in degeneracy and near-degeneracy, among them. In ergodic systems, on the other hand, there is found a kind of repulsion between nondegenerate energy levels that makes the spectrum smooth and regular rather than noisy. This is in full agreement with the “level repulsion” seen in perturbation theory [41] in response to additional coupling.

The spin and angular momentum conservation of electron motion in atoms and atomic ions introduces dynamical decomposition and leads to highly nonergodic energy spectra. This is most easily seen for single electron hydrogen and atomic ions where the degeneracy is g(*n*) = 2*n*^2^ with the principal quantum number *n* = 1, 2, … ∞. As we add electrons, the degeneracy expands due to the greater number of degrees of freedom, but at the same time, it contracts due to symmetry breaking by the electron-electron repulsion and spin-orbit coupling. While the total degeneracy may then decrease, the coupling introduced is often weak (particularly the spin-orbit coupling), acting only as a minor perturbation so that some degeneracies split up merely to form near-degeneracies, which, as noted above, have much the same effect on the atomic reactivity.

All atoms show nonergodic electron motion with degeneracies and near-degeneracies, but the question is whether this applies to the ground state and the nearest excited states. If it does, the atom is expected to be reactive. If not, it will be nonreactive. It is important to note now that the inert gas atoms have nondegenerate ground states and that the energy gaps to electronic excited states are large. Thus, they respond weakly to external perturbations. This is an important, we claim the decisive, reason for their lack of reactivity. Nonergodicity in the form of ground state degeneracy and near-degeneracy, on the other hand, makes the electronic structure of a reactive atom responsive to various types of coupling and to the chemical bonding mechanisms like the transfer and sharing mechanisms visualized first by Lewis [45] and Langmuir [46], and later adopted, and still used, by most chemists.

**Simplified analysis of degeneracy in the Aufbau picture:** In order to see whether this association of atomic reactivity with ground state degeneracy or near-degeneracy can be found in reality, we shall use a simplified analysis based on the empirical and qualitative quantum mechanics of the Aufbau picture of atoms. It is used in most introductory chemical text books to explain the periodic table as a consequence of ground state electronic structure of the atoms [1,8,9,11]. The Aufbau picture is an empirical extension of the hydrogenic model of atoms in which the electrons do not interact by Coulomb repulsion but obey the Pauli principle and “rules” that empirically account for electron–electron repulsion. First the electron–electron repulsion is added in the form of screening of the electron–nucleus attraction. This leads to an orbital energy dependence on both the prime and the angular momentum quantum numbers *n* and *l*, respectively. The relevant energy ordering of these subshell is 1s, 2s, 2p, 3s, 3p, and 4s and is introduced empirically. Hund’s rules then guide the occupation of orbitals to avoid doubly occupying orbitals which are in the same subshell and maximally align spins when orbitals are singly occupied. This qualitative quantum mechanics is normally only used to identify atomic ground state structures, but we shall use it also to describe ground state degeneracy.

**Figure 5:**
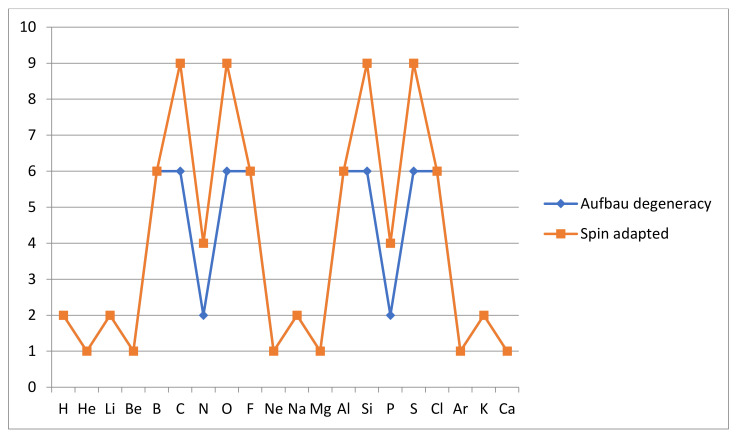
Aufbau degeneracies are compared with degeneracy of spin-adapted configuration combinations for the ground states of the first twenty atoms H, He, … Ca. Deviation is seen for C, N, O and Si, P, S. For other atoms Aufbau degeneracy is not changed by spin adaptation.

Note that the Aufbau picture is a one-electron picture in the sense that it is assumed that there is a one-electron Hamiltonian which describes the many-electron environment so that its corresponding eigenstates of spin-orbitals can be used to construct correspondingly many-electron eigenstates as determinants formed from the chosen canonical spin-orbitals. This picture is used in Hartree-Fock theory of atomic structure but is more general and could be based also on density functional theory. It should be noted that the Aufbau configurations are atomic energy eigenstates only in this independent-electron approximation but can be regarded as the most effective basis configurations in the true many configuration ground states which include the effects of electron correlation and spin-orbit coupling. The effectiveness of such basis configurations not only in the atomic ground state calculation but in resolving the response to external perturbations and in molecule formation, is dependent on their energy, which should be as low as possible. Thus, the degeneracy analysis to follow can be understood as an attempt to identify the number of highly effective basis configurations a certain atom brings with it to the process of molecule formation in a full multi-configurational description. For those familiar with the standard fully spin-coupled electron analysis of atomic ground states, it should be noted that the Aufbau analysis below is generally, but not always, in agreement with the exact analysis of degeneracy. Spin adaption by a combination of small sets of two or three of our Aufbau configurations recovers two or three additional ground states from the nearly degenerate Aufbau configurations in the case of atoms C, N, O and Si, P, S, as shown in Figure 5 below.

The Aufbau rules (see reference [11] Section 2.18) for atomic ground states, which allow atomic configurations in a mean field (or SCF) approximation to be energy-ordered for the purpose of selecting a minimum, the ground state, have a natural degeneracy of the ground state g_0_(A). It arises because more than one spin-orbital configuration satisfies all the applicable rules. There may be further coupling (e.g., spin-orbit coupling) that would split such a degeneracy, but it is expected to be weak and merely to turn a degeneracy into a near-degeneracy with a similar effect. As shown below, we have no degeneracy for the inert gases, i.e., g_0_(He) = g_0_(Ne) = g_0_(Ar) = 1, while all other atoms among our first 20, except Be, Mg and Ca, have degenerate Aufbau ground states as an indication of reactivity. We now examine these degeneracies, and the likely near-degeneracies, that may explain the observed reactivity of Be, Mg and Ca.

**The Aufbau rules and their simple interpretation are as follows**: The spin orbitals are denoted *n, l, m_l_* and *m_s_* with *n* = 0, 1, …∞, *l* = 0, …*n* − 1, *m_l_* = −l, …l, *m_s_* = ±½ and are singly occupied or unoccupied with the main subshell (*n,l*) energy ordering 1s, 2s, 2p, 3s, 3p, 4s, and 3d. This ordering is related to the screening of the electron-nucleus interaction by the repulsion from the “other” electrons.

The spatial orbitals are denoted *n, l, m_l_*. There are 2*l* + 1 in each subshell. They are of equal energy (degenerate), but with more than one electron in the subshell a) the orbitals should remain singly occupied as much as possible and b) when two or more are singly occupied, the spins of the electrons in them should all be aligned up (*m_s_* = +½) or down (*m_s_* = −½). Both these rules, 2a and 2b, relate to electron–electron repulsion beyond the mean field screening accounted for in rule 1. By 2a, electrons are kept spatially apart and by 2b, stabilizing exchange effects are brought in due to wave function antisymmetry.

Note that these rules, the subshell ordering (1) and Hund’s rules (2), are applied sequentially, but there are cases, although not among the first 20 atoms that we consider here, where in reality rule 2 interferes with rule 1. As noted, these rules form a kind of empirical quantum mechanics for independent electrons in atoms. In our qualitative discussion of the relationship between atomic ground state degeneracy and reactivity, we shall employ these familiar Aufbau rules. Their lack of rigor is at least partially compensated by the fact that the effects we are looking for are present and are continuously variable with the energy splitting among the degenerate or near-degenerate states. As examples, we note that H has g_0_(H) = 2 since the rules leave the spin of the electron to be either up (+½) or down (−½), while for oxygen, four 2p-electrons mean three possibilities of doubly occupying a 2p-orbital and then the spins of the two electrons singly occupying the other 2p-orbitals can either both have a spin up or down, which yields g_0_(O) = 3 × 2 = 6. The variation of the ground state degeneracy according to the Aufbau rules is shown in Figure 5 below. The traditional spin adapted ground state degeneracy is included. Note that when only one curve is shown, the two methods (Aufbau and spin adapted) agree on ground state degeneracy.

The results show eight strongly and six weakly degenerate atoms of g_0_ = 6 and 2 in the Aufbau representation, respectively, while the remaining six atoms have nondegenerate Aufbau ground states. This is both expected and unexpected. Most of the atoms, except for the three inert gas atoms, show significant degeneracy of the ground state. In particular, intuitively strongly reactive atoms like B, C and O, F in the first row and Al, Si and S, Cl in the second row, all have a degeneracy of 6. H, Li, N and K have a double degeneracy. However, the inert gas atoms He, Ne and Ar are not the only atoms with nondegenerate ground states. Be, Mg and Ca are also nondegenerate, which may appear counterintuitive. The interesting question arises: What is the origin of the apparent inertness, seen from a perspective of degeneracy, of beryllium, magnesium and calcium? Our discussion above suggests that the answer will be found in the further consideration of “near-degeneracy”. Note that the consideration of near-degeneracy also will reduce the difference between the Aufbau and spin-adapted representations of atomic states, since the added ground states in the spin-adapted representation will be appearing as near-degenerate in the Aufbau representation.

The Aufbau rules assign a nondegenerate 1s^2^2s^2^ to Be, 1s^2^2s^2^2p^6^3s^2^ to Mg and 1s^2^2s^2^2p^6^3s^2^3p^6^4s^2^ to Ca due to the energy ordering of the subshells as 1s, 2s, 2p, 3s, 3p, 4s etcetera. We note that the shells are defined by the principal quantum number *n* = 1, 2, 3, 4 etcetera. If we grossly simplify the treatment of electron–electron repulsion and assume that the effective screened potential has the form of a central Coulomb potential with an effective charge Z_eff_e accounting for the screening, then 2s and 2p orbitals, as well as the 3s and 3p orbitals, are degenerate since only the principal quantum number n determines the orbital energies. Under those conditions, the ground states of beryllium and magnesium would be highly degenerate. The breaking of this degeneracy is due to the deviation of the s and p energies from the same shell, where the s-orbitals are of lower energy since they penetrate more deeply into the core region where the effective charge is less screened. Particularly for the atoms like Li and Be or Na and Mg with only one or two electrons in the new shell, the s-p splitting is likely to be small and the ground states then become near-degenerate, i.e., close in energy, and reactive. The inert gas atoms, on the other hand, have no degeneracy or near-degeneracies and consequently become nonreactive.

**Near-degeneracy due to small ns-np splitting:** Our conclusion is that ground state degeneracy according to the Aufbau rules is closely related to atomic reactivity and can help in the interpretation of the reactivity measures AR_M_(A) and AR_P_(A). The fact that the ns-np splitting is small implies that at least for n = 2 the transfer of electrons from s to p subshells in the same shell can be assumed to yield near-degeneracy of consequence for atomic reactivity. For n = 3 and with the increasing number of electrons, the s-p splitting increases and in the case of n = 4 and higher this type of near degeneracy is likely to be not “near” enough to be of consequence. It is not difficult to work out the number of nearly degenerate states due to small s-p splitting in the case of the light atoms. Note that in the analysis that follows, all other Aufbau rules are adhered to, except that shifts of electrons from 2s to 2p orbitals are permitted. The number of states nearly degenerate with the ground state due to s to p shifts is called ng-sp and we find it as follows. For H and He, there is no such degeneracy possible, but for Li, the 2s electron may be transferred to three 2p-orbitals and the spin may be up or down, which leads to ng-sp = 6. For Be, we have six states with one electron shifted to a 2p-orbital and six states with two electrons so shifted with all spins aligned. Thus, we find that ng-sp = 12 for beryllium. Note that if there is an energy split for the 2s→2p shift ΔE_2sp_ > 0 then shifting of two electrons will incur a double energy penalty and such configurations are of less impact on reactivity, but our analysis is qualitative, so we do not account for this. For boron B we have 2s^2^2p^1^ for the valence electrons according to the Aufbau rules, but allowing transfer from 2s to 2p we get eight states with only single orbital occupation, so we get ng-sp = 8. For carbon C we have two states of four singly occupied 2s- or 2p-orbitals making ng-sp = 2. For nitrogen N we cannot shift an electron from 2s to 2p without doubly occupying the higher orbital so we have ng-sp = 0. Again, for oxygen O we cannot shift an electron from 2s without doubly occupying another 2p-orbital, so ng-sp = 0 and the same holds for fluorine F.

The results quoted here apply also to the second row atoms with respect to a corresponding 3s-3p splitting which, however, is generally considerably larger. Thus, we must expect the corresponding near-degeneracies to have less of an effect on the reactivity of these heavier atoms. We note that the s-p near-degeneracies seem to have the largest effect on the light first row atoms Li, Be, B and C. There are, of course, further s-p shifted excited states that could be considered, e.g., those including new doubly occupied p-orbitals. They are expected to be higher in energy and not quite so “near” degeneracies and we will not consider them here.

**Hund’s rule near-degeneracy:** Another type of near-degeneracy of the atomic ground state in the Aufbau picture likely to be important is due to the relaxation of Hund’s rule maximal spin alignment of electrons in singly occupied orbitals. Given that this rule has an effect only for electrons singly occupying several spatially different orbitals, we can expect that the energy required to flip these spins will be relatively small given that the Fermi hole creation around spin-aligned electrons due to wave function antisymmetry has an effect only in proportion to the spatial overlap of the orbitals. The corresponding number of nearly degenerate states obtained by spin flipping will be called ng-H. Note that we again obey all other Aufbau rules, including s-p subshell ordering followed by minimal orbital occupancy, in working out this number. The first atom to show such a spin flip near-degeneracy is carbon C, where the two aligned 2p electrons can now be one up and one down but still in different 2p-orbitals. There will be six such states, so we have ng-H = 6 for carbon. For nitrogen N we can have two up and one down or one up and two down, which together yields ng-H = 6. For oxygen O, we can flip one spin in a singly occupied 2p-orbital and counting all ways to do this we get ng-H = 3 × 2 = 6. Fluorine F does not rely on any Hund’s rule alignment, so we have ng-H = 0 as was the case for the first five atoms. The same arguments apply to the second row, so we have ng-H = 0 except for Si, P, S where it is 6, 6 and 6, respectively. We note that nitrogen has Hund’s rule near-degeneracies, which might help explain why it appears to be rather reactive according to the AR_GP_ measure, despite a low AR_M_ reactivity and degeneracy. In fact, it is not difficult to see that the Aufbau near-degeneracies for nitrogen includes the extra two degeneracies achieved in the spin-adapted set of degenerate ground states. Thus, the inclusion of near-degeneracy reduces the apparent difference between the Aufbau and the spin-adapted approach to atomic ground and first excited states.

**Figure 6:**
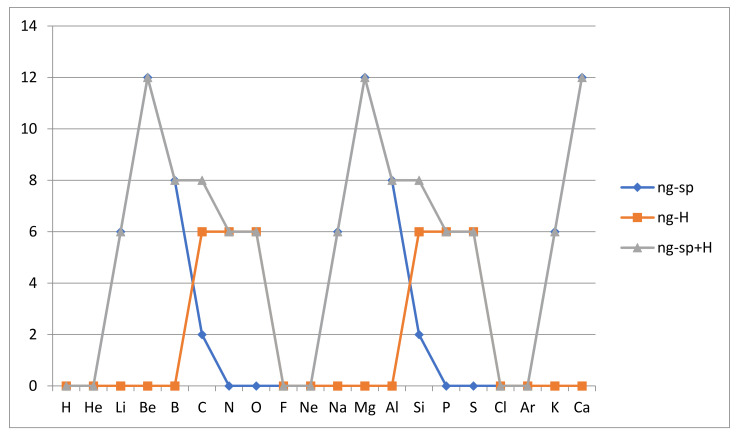
The variation in s-p and spin flip (Hund’s rule) near-degeneracy of the Aufbau ground state is shown for the atoms H through Ca. The sum of these near-degeneracies ng-sp + H = ng-sp + ng-H is also shown.

The s-p and Hund’s rule near-degeneracies in the Aufbau picture are summarized and illustrated in Figure 6 below.

We see that the claimed role of Aufbau near-degeneracy would readily explain why Be, Mg and Ca are still reactive despite having nondegenerate Aufbau ground states. They all have a strong s-p near-degeneracy of 12. The “degree of near-degeneracy”, i.e., where “near” is defined by the energy gap to the ground state energy, should be highest for Be, smaller for Mg and smaller still for Ca. The weak degeneracy of N and P of g_0_ = 2 is also complemented by Hund’s rule near-degeneracy of 6, which helps explain why these atoms are quite reactive.

The key insight is that ground state degeneracy is closely related to atomic reactivity in a qualitative way, but should be complemented by the effect of near-degeneracies to become more reliable in explaining quantitative variation of atomic reactivity. The underlying physical origin of this connection between ground state degeneracy and reactivity is that both reflect dynamical constraints, which strain the atom in proportion to the degree of degeneracy. These strains in the bonding atoms then provide the source of stabilization as they are relaxed by chemical bonding in molecule formation. Evidence of the role of degeneracy in this process can readily be found. We know, for example, that promotion and hybridization in empirical VB theory are based on shifts of electrons from 2s to 2p in order to explain the geometries of small molecules. This is clearly related to the s-p near-degeneracy discussed above. The fact that this type of hybridized geometry reverts towards an unhybridized form for heavier central atoms is, of course, related to the wider spread of 3p from 3s orbital energies as noted above. This reminds us that the importance of near-degeneracy is in inverse proportion to the relevant energy gap between ground and excited state. The consideration of the physical basis of the Aufbau rules allows us only to crudely estimate the magnitude of these gaps. The simple degeneracy analysis above is therefore only qualitative and suggestive. There is useful work to be done by more accurate methods to further delineate the role of degeneracy, exact or near, in atomic reactivity.

**Direct estimation of atomic reactivity—Thomas-Fermi DFT as a nonreactive reference**

Finally, in our analysis of the origin and appropriate measure of atomic reactivity, we shall consider whether it would be possible to estimate the reactivity directly by quantum calculation of the atomic ground state, i.e., with reactivity turned on or off in otherwise equivalent quantum calculations. This is a very fundamental question and it may not be definitively answerable without a better understanding of reactivity than what is presently available. It turns out, however, that a very approximate approach of this sort is possible and has been tried in earlier work [32,33]. It is based on the use of the Thomas-Fermi theory of electronic structure which, despite capturing many important features of quantum mechanics, was found to be completely unable to resolve covalent bonding [28,29]. Thus, it is possible to use this early form of density functional theory (TF-DFT) as a “nonreactive reference theory” and compare its results with proper quantum chemical results by a method known to be able to predict reactivity with reasonable accuracy. The analysis that follows uses a semiclassical form of quantum mechanics, which includes TF-DFT, implemented approximately, but nevertheless we suggest that it provides a useful insight into the strain energies of atoms. In essence, this analysis amounts to an interpolation of accurate quantum chemical ground state energies of He, Ne and Ar to yield ground state energies for all 18 atoms from H to Ar in an “inert gas atom model”.

The “density functional theory” (DFT) of an electronic structure started with the Thomas-Fermi theory (TF-DFT) of nearly 100 years ago [26,27]. It is a true, or “orbital free”, form of DFT, which only requires the electron density ρ(**r**), assumed unpolarized [47]. It is therefore comparatively simple to handle either by analytical mathematics or by simple numerical methods. The functional for the electronic energy of an atom is

E(ρ) = C∫d**r**ρ^5/3^(**r**) − ∫d**r**ρ(**r**)Ze^2^/|**r**| + ½ ∫∫d**r**d**r’**ρ(**r**)ρ(**r’**)e^2^/|**r** − **r’**| (13)

with C = (3h^2^/40m) × (3/π)^2/3^. The Thomas-Fermi theory incorporates a great deal of basic quantum mechanics but, unfortunately, this simple functional, which on the RHS has terms for, in order of appearance, the kinetic energy, the electron-nucleus attraction and the electron–electron repulsion, also suffers from some major errors. The highly successful modern form of DFT owes much to the simple TF-DFT but has seen a major influx of “orbital dependence” from the Hartree–Fock theory in arriving in its modern form [3,48].

The most obvious flaw of TF-DFT may be the fact that the last term in the functional in (13) lacks the exchange effect due to the asymmetry of the electronic wave function and includes electron–electron self-interaction. The main error, however, is in the kinetic energy term, first on the RHS, which is of a semiclassical character and is local in space. Upon closer examination, it is soon realized that it fails to account for the strong quantum mechanical coupling between the dynamics of the electrons and their correctly quantized kinetic energy. In fact, it is readily seen that this term is insensitive to whether parts of space integrated over are dynamically connected or not and therefore TF-DFT is unable to resolve what we have claimed to be the main mechanism of covalent stabilization in molecule formation [25,31,49].

The complete failure of TF-DFT to resolve covalent bonding was first proven by Teller [28] and Balazs [29]. The origin of this failure, i.e., the inability to account for the role of dynamics in quantization, has been known for some time [25] and a number of ways to incorporate dynamical constraints have been explored [30,50]. It has thus been found that TF-DFT provides an “inert gas approximation” for atomic electronic structures, which makes the atoms nonreactive by effectively treating the dynamics as if it were fully coupled and ergodic. There is no shell and subshell structure in the TF model of the atom, but such structure can be reintroduced by extension of the model. Thus it has been found to be possible to mimic the conservation of spin and angular momentum in the TF-quantization of atomic structure [32,33,50]. The calculation of a ground state energy and electron density could then be carried out with and without these conservation laws acting to constrain the quantization and the cost in energy of the constraints identified as the reactivity of the atom. The calculations were done with a corrected form of TF-DFT and used simple exponential electron densities for each shell [32,33]. It did not resolve the s-p energy splitting in the L and M shells but corrected for self-interaction among the electrons as well as for the gradient contribution to kinetic energy according to von Weizsäcker [51], scaled so as to get accurate energies for the inert gas atoms. Thus, one could say that the revised TF-DFT calculation was used as an interpolation method to indicate what ground state energies an inert gas atom model would yield for the reactive atoms. The resulting estimates of the cost in energy associated with the spin and angular momentum constraints fall in a range from a maximum of 3910 kJ/mol for oxygen to 630 kJ/mol for hydrogen and zero for the inert gas atoms. Clearly, such energies are more than sufficient to be the main contributors to the bond energies of molecules. Indeed, a full relaxation of these dynamical constraints on atoms is not achieved in bonding. There are antibonding mechanisms, primarily repulsion between nuclei and Pauli repulsion between nonbonding electrons, which prevent the full strain energy of reactive atoms to be recovered as bond energy. Typically, only about a third of this estimated strain energy is seen as bond energy in hydride molecules.

**Figure 7:**
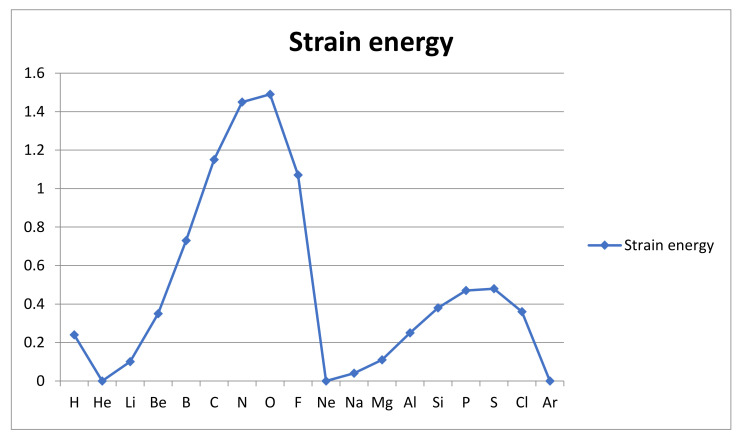
Atomic strain energy due to conservation laws are shown in a. u. (1 a. u. = 2626 kJ/mol). The estimates for the atoms H through Ar are obtained from a simplified and extended form of Thomas-Fermi theory. See references [32,33,50] for more detail.

The estimated total atomic strain energies, obtained by an approximate insertion of conservation laws into a simplified TF-DFT calculation, are shown in Figure 7 below.

The estimated atomic strain energy shows a simple periodic pattern with minima of zero at the inert gas atoms. There is a slow rise in the beginning of the two periods followed by a sharp fall at the end. We see that the maximal strain energy arises for oxygen at 1.49 a.u., closely followed by nitrogen at 1.45 a.u. The pattern is similar in the second period, but the strain energy is scaled down by about a factor of 1/3. The major trend within a shell seems to be that the strain increases with the nuclear charge Ze, but the natural valence number is also important, e.g., in making double bonding oxygen more reactive than single bonding fluorine despite the higher nuclear charge of fluorine. The strain energy is clearly a multi-electron measure rather than a one-electron or one single bond measure like AR_M_ and AR_GP_, respectively. Nevertheless, the strain energy correlates rather well with our preferred atomic reactivity measure AR_GP_. It is noteworthy also that the second period (M-shell) atoms are less strained (and presumably less reactive) than those from the first full period.

These trends, seen in the atomic strain, fit well with the observed tendencies in bond strengths and molecular stabilities. We should note that while the strain energies are of a magnitude similar to the Mulliken reactivities, they are about half an order of magnitude larger than the typical single bond energies or Pauling reactivities. Clearly, the atomic strains are only partially relaxed in molecule formation by covalent bonding. Even very stable molecules still carry an atomic strain due to conservation laws and local decoupling of electron motion around atomic centers. We must also note that there are also molecular strains due to antibonding mechanisms which cancel some of the stability contributed by the relaxation of atomic reactivities. The repulsion between nuclei and between nonbonding electrons eventually balance the attractive relaxation of atomic strains as the molecule forms. Thus, compared to the total strain in the separate atoms, the molecular binding energy is only something like a third as large. The strain has been converted to molecular binding energy by the delocalized motion and redistribution of valence electrons within the molecule. Part of it has also been used to overcome the repulsion between nuclei and Pauli repulsion.

## 4. Discussion and Conclusions

The atoms are the building blocks of molecules and substances and thereby also of the discipline of chemistry itself. The atomic elements depicted in the form of the periodic table serve as an informal flag to mark a room as devoted to chemistry. The most important property of an atom is the reactivity which, to a variable degree, drives two or more of them to combine into molecules and aggregate them into substances. In an intuitive and empirical sense, the reactivity of atoms is also their best known property, with its variation summarized to the initiated in the periodic table, but, oddly, the discussion of the origin and magnitude of their reactivity seems not to have included a measure of covalent reactivity. There is a related but, we have claimed here, significantly different, concept of electronegativity and corresponding measures by Pauling [6] and Mulliken [7], later modified and extended in many ways, which have long been a central part of chemistry used to understand and predict the preferred bond type and bond polarity in chemical bonding.

Given the popularity and common use of electronegativity, also for matters closely related to reactivity, justification is needed for our proposed addition of the concept of atomic reactivity. The most immediate justification is that atomic reactivity is undeniably important and distinctly different from electronegativity. The “power of an atom in a molecule to attract electrons” is not sufficient to describe atomic reactivity, nor is the mechanism of charge redistribution the primary feature of the interaction of reactive atoms in a stable molecule. A more important feature of an atom in a molecule is the “partial occupation of atomic orbitals of the same or similar energy”. Thus, spin-orbitals, which in the single atom ground state were degenerate or near-degenerate, but some occupied and others not, may in the molecule be more uniformly but partially occupied. In the case of the hydrogen atom used in our definition of Pauling-like AR_P_ and AR_GP_ measures, the atom in the hydride molecule may be charged to some degree, as in the ionic bonding mechanism, but more importantly, it is always unpolarized with respect to spin, i.e., the two degenerate atomic spin states are equally occupied for electrons around the hydrogen atomic center in the molecule. Thus, the interatomic electron transfer in the molecule has had the effect of partially and uniformly occupying both H1s↑ and H1s↓ atomic orbitals, if a minimal basis set is used for the hydrogen atom. This brings the local environment around the H atom to a helium-like character by partial spin-orbital occupation, which stabilizes the hydrogen atom and this stability is part of the binding energy of the molecule.

Similarly, larger atoms can be brought towards an inert gas-like electronic structure by the type of “partial orbital occupation”, resulting from interatomic electron transfer. In Hartree–Fock or DFT calculations, the interatomic electron transfer is seen in the formation of delocalized molecular orbitals, but the same effect can also be achieved in correlated calculations. The Heitler–London wavefunction for H_2_ is a particularly clear example of how two polarized configurations, by addition, represent the unpolarized hydrogen atom environments in the H_2_ molecule, but it should be noted that only when the electrons are able to oscillate rapidly between the atoms is the stabilization associated with partial occupation realized [49].

Motivated by the general considerations above, our aim here has been twofold: (i) to revise the Pauling and Mulliken measures of atomic electronegativity so that they apply broadly to atomic reactivity expressed in covalent bonding instead, and (ii) to identify the quantum mechanical origin of the reactivity of all atoms except the inert gas atoms. Three different empirical measures of atomic reactivity have been put forward. The measure AR_M_ is based on a modification of the Mulliken measure of electronegativity and uses atomic ionization energies. The other two measures are based on the type of reasoning applied by Pauling in analyzing electronegativity in terms of bonding, in his case an ionic contribution to bonding. Atomic hydrides have been used to extract reactivities from molecular stabilities, assuming first a linear algebraic average, and then a geometric average relationship. The latter leads to a particularly useful atomic reactivity measure closely related to bond energy and displaying a simple periodic variation. It should be noted that both these Pauling-like measures relate most directly to the covalent bonding mechanism and do not specifically account for the ionic contribution used by Pauling in his work on electronegativity. Explicit reference to the ionic bonding mechanism could be added but only at the cost of a loss of simplicity. It is interesting, therefore, to note that the Pauling type of electronegativity, which is based directly on the ionic bonding mechanism, nevertheless correlates strongly with our proposed atomic reactivity AR_GP_, as shown in Figure 4.

Finally, the underlying cause of atomic reactivity is examined and found in the dynamical constraints acting on the electrons in the presence of a spherically symmetric attraction to the nucleus. The resulting shell structure, well known to chemists in the Aufbau picture, is the origin of the periodic variations of atomic properties including reactivity, which is found to be related to incomplete coupling of the electronic motion. Thus, we can relate reactivity qualitatively to the Aufbau ground state degeneracy and near-degeneracy. The quantitative explanation of the total strain imposed by these dynamical restrictions is hidden in the depth of quantum mechanics, but a very approximate, but still quantitative, analysis is available in earlier work [32,33]. Employing an extension of the Thomas-Fermi theory, the strain energy of atoms, due to the dynamical constraints associated with the shell structure of the atoms, could be turned on and off. The total atomic strain energy varies periodically in this estimate much like the reactivity but is much larger. This difference in magnitude follows, since, as could be expected due to antibonding mechanisms, only a minor part of the strain is turned into binding energy in molecule formation by covalent bonding.

We conclude that atomic reactivity can be usefully estimated quantitatively but with a proviso applicable also to electronegativity, i.e., atomic reactivity can be expected to defy unique definition. It is a complex multidimensional property expressed in bonding and molecule formation to a variable degree. Reduction to a one-dimensional numerical value cannot be done in a definitive and unique way. A full exploration, seeking verification or falsification, of the usefulness of the above-proposed measures, is not offered here. In the case of measures of electronegativity, this process is still going on. The related concept of atomic reactivity may be no easier to scrutinize, but we are confident that the Pauling-like measure AR_GP_, based on the assumption that a bond energy is proportional to a geometrical average of the two atomic reactivities, will readily be found to be helpful. The gain in understanding of the origin of atomic reactivity should be equally apparent. We have shown how it follows from basic features of quantum mechanics relating to the motion of electrons in atoms. The decisive role of dynamical constraints and corresponding degeneracy or near-degeneracy among the atomic energy eigenstates can be understood within the familiar Aufbau picture of an atomic structure and explored with the aid of the simple original Thomas-Fermi form of DFT.

## Figures and Tables

**Table 1:** First ionization energies and reactivity measures for atoms H, He and Ca developed according to reasoning of Mulliken (AR_M_) and Pauling (AR_P_). Note that Z is the atomic number and the inert gas atoms have only nominal reactivities AR_P_ and rAR_P_ based on vanishing atomization energies for the dimers HeH, NeH and ArH.

Atom	Z	I_1_(Z) kJ/mol	I_1_(Z + 1) kJ/mol	AR_M_ kJ/mol	AR_P_ kJ/mol	rAR_M_	rAR_P_
H	1	1310	2370	530	230	10.6	2.55
He	2	2370	519	−926	−230	−18.5	−2.55
Li	3	519	900	191	15	3.82	0.17
Be	4	900	799	−51	163	−1.02	1.81
B	5	799	1090	146	486	2.92	5.39
C	6	1090	1400	155	838	3.10	9.29
N	7	1400	1310	−45	553	−0.90	6.13
O	8	1310	1680	185	511	3.70	5.66
F	9	1680	2080	200	361	4.00	4.00
Ne	10	2080	494	−793	−230	−15.9	−2.55
Na	11	494	736	121	−37	2.42	−0.41
Mg	12	736	577	−80	−16	−1.60	−0.18
Al	13	577	786	105	198	2.10	2.19
Si	14	786	1060	137	431	2.74	4.78
P	15	1060	1000	−30	316	−0.60	3.50
S	16	1000	1260	130	302	2.60	3.35
Cl	17	1260	1520	130	213	2.60	2.36
Ar	18	1520	418	−551	−230	−11.0	−2.55
K	19	418	590	86	−55	1.72	−0.61
Ca	20	590	632	21	1	0.42	0.01

**Table 2:** Average bond energies (E_b_(AH)) and geometric Pauling-like atomic reactivities AR_GP_ in kJ/mol together with corresponding relative reactivities rAR_GP_ are shown for the atoms H through Ca.

Atom (A)	Molecule	E_av_(AH) in kJ/mol	AR_GP_(A) in kJ/mol	rAR_GP_(A)
H	H_2_	460	230	2.42
He	HeH	0	0	0
Li	LiH	245	65	0.68
Be	BeH_2_	312	106	1.12
B	BH_3_	392	167	1.76
C	CH_4_	440	210	2.21
N	NH_3_	414	186	1.96
O	H_2_O	486	257	2.71
F	HF	591	380	4.00
Ne	NeH	0	0	0
Na	NaH	193	40	0.43
Mg	MgH_2_	222	54	0.56
Al	AlH_3_	296	95	1.00
Si	SiH_4_	338	124	1.31
P	PH_3_	335	122	1.29
S	H_2_S	381	158	1.66
Cl	HCl	443	213	2.25
Ar	ArH	0	0	0
K	KH	175	33	0.35
Ca	CaH_2_	216	51	0.53
